# The role of histone methylation in the development of digestive cancers: a potential direction for cancer management

**DOI:** 10.1038/s41392-020-00252-1

**Published:** 2020-08-03

**Authors:** Yuan Chen, Bo Ren, Jinshou Yang, Huanyu Wang, Gang Yang, Ruiyuan Xu, Lei You, Yupei Zhao

**Affiliations:** Department of General Surgery, Peking Union Medical College Hospital, Chinese Academy of Medical Sciences, Peking Union Medical College, 100023 Beijing, PR China

**Keywords:** Cancer genomics, Cancer genetics, Gastrointestinal cancer, Gastrointestinal cancer, Drug development

## Abstract

Digestive cancers are the leading cause of cancer-related death worldwide and have high risks of morbidity and mortality. Histone methylation, which is mediated mainly by lysine methyltransferases, lysine demethylases, and protein arginine methyltransferases, has emerged as an essential mechanism regulating pathological processes in digestive cancers. Under certain conditions, aberrant expression of these modifiers leads to abnormal histone methylation or demethylation in the corresponding cancer-related genes, which contributes to different processes and phenotypes, such as carcinogenesis, proliferation, metabolic reprogramming, epithelial–mesenchymal transition, invasion, and migration, during digestive cancer development. In this review, we focus on the association between histone methylation regulation and the development of digestive cancers, including gastric cancer, liver cancer, pancreatic cancer, and colorectal cancer, as well as on its clinical application prospects, aiming to provide a new perspective on the management of digestive cancers.

## Introduction

Digestive cancers, including gastric cancer (GC), liver cancer, pancreatic cancer (PC), colorectal cancer (CRC), etc., are commonly observed malignancies in clinical practice. The latest epidemiological data indicate that an estimated 333,680 people will be diagnosed with digestive cancers in the United States in 2020 and that ~167,790 will die of these diseases; both of these estimates rank first among all cancers.^1^ Although progress has recently been made in the management of digestive cancers, the 5-year survival rate of some cancers remains unsatisfactory—for example, 18% for liver cancer and only 9% for PC.^1^

Therefore, further study of the molecular mechanism underlying digestive cancers is urgently needed. The interest in epigenetics has increased. Histone modifications, including acetylation, methylation, phosphorylation, and ubiquitylation, are a pivotal form of epigenetic information. Each of these modifications is associated with gene activity, gene silencing, or insulation between active and inactive gene regions.^2^ For example, the transcription factor FOXA1 drives large-scale enhancer reprogramming through H3K27ac (acetylation of lysine 27 of histone 3) modulation, thereby promoting PC progression.^3^ An increase in H3K9me2 in the promoter region of RARRES3 gene represses its transcription, thereby contributing to cancer cell migration in hepatocellular carcinoma (HCC).^4^

**Fig. 1:**
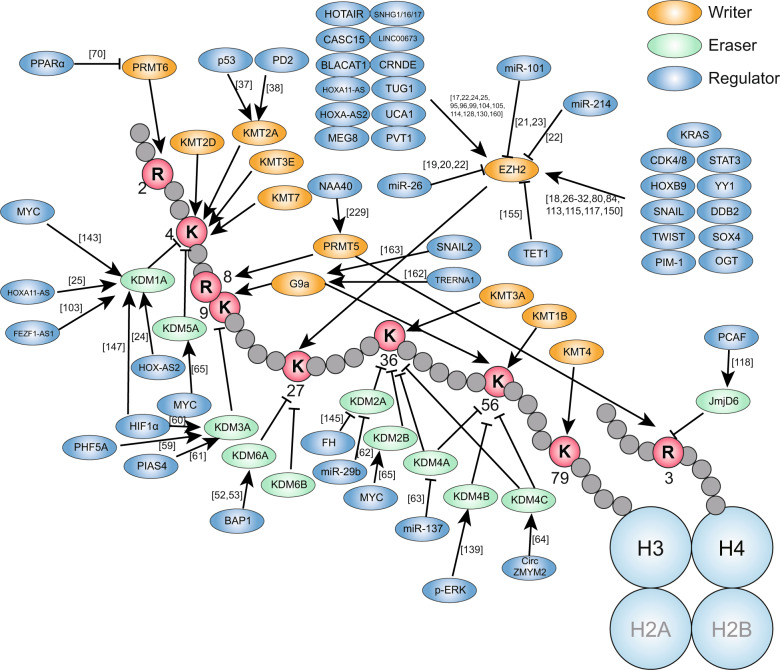
Activators or inhibitors of histone methylation modifiers in digestive cancers. During the development of digestive cancers, regulators activate or inhibit histone methylation modifiers (writers and erasers, which add methyl groups to or remove methyl groups from histone lysine residues, respectively), and ultimately regulate the expression of cancer-related genes

**Fig. 2:**
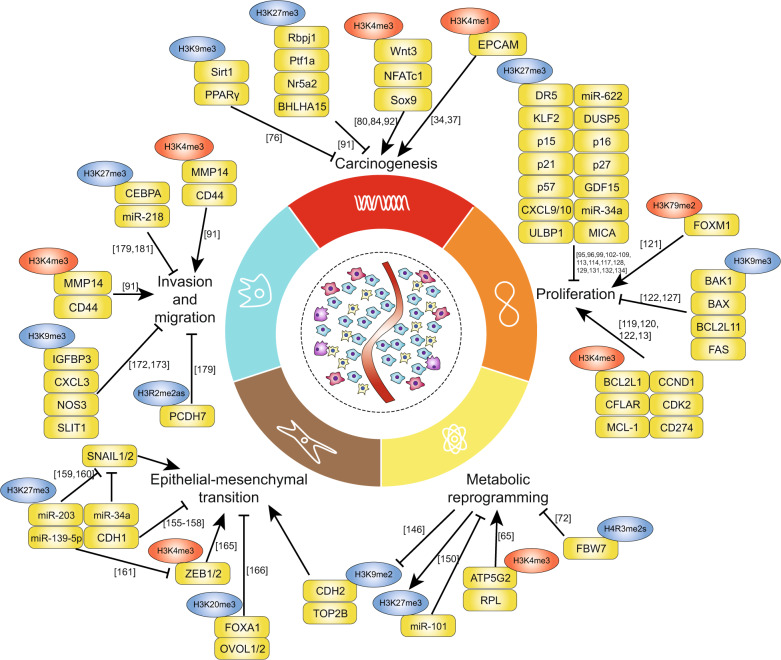
Regulation of the histone methylation of tumor-associated genes during digestive cancer development. Under certain conditions, aberrant methylation or demethylation of the corresponding cancer-related genes contributes to different processes and phenotypes during digestive cancer development, including carcinogenesis, proliferation, metabolic reprogramming, epithelial–mesenchymal transition, invasion, and migration. The blue circles indicate modifications that downregulate gene expression, while the red circles indicate modifications that upregulates gene expression

Since extensive studies have recently revealed the key role of histone methylation regulation in the development of digestive cancers, this review focuses on histone methylation. We highlight a cluster of histone methylation modifiers that play a vital role in digestive cancers—KMTs (lysine methyltransferases), KDMs (lysine demethylases), and PRMTs (protein arginine methyltransferases) (Fig. 1). These modifiers cause gene silencing or activation through the methylation or demethylation of related genomic histones, playing a crucial role in aspects of digestive cancer development, such as carcinogenesis, proliferation, metabolic reprogramming, epithelial–mesenchymal transition (EMT), invasion, and migration (Fig. 2). This review aims to discuss the association between the regulation of histone methylation and the development of digestive cancers, including GC, HCC, PC, and CRC, and to consider the underlying mechanism. This knowledge may provide a new perspective on the management of digestive cancers.

## Main modifiers of histone methylation in digestive cancers

### KMTs

KMTs are a group of enzymes that catalyze monomethylation, dimethylation, or trimethylation by adding one, two, or three methyl groups, respectively, from S-adenosyl-l-methionine to the ε-amino group of a histone lysine residue, thus regulating gene expression. For example, H3K27me3 (trimethylation of lysine 27 of histone 3) is often associated with transcriptionally repressed chromatin, and H3K4me3 is often linked to transcriptionally active chromatin. According to their defined protein domain or homologous sequence, KMTs are classified into eight distinct subfamilies: KMT1–8.^5^

A cluster of KMTs, including the H3K36 methyltransferase KMT3A,^6^ the H3K9/56 methyltransferase G9a (also called KMT1C),^7^ the H3K27 methyltransferase enhancer of zeste homolog 2 (EZH2, also called KMT6A),^8,9^ and the H3K4 methyltransferases KMT3E,^10,11^ KMT2A,^12,13^ KMT2D,^14^ etc., has been found to be ectopic expressed in digestive cancers. Among these KMTs, EZH2, a catalytic subunit of polycomb repressive complex 2 (PRC2),^15^ is one of the most commonly reported methyltransferases that represses gene expression in digestive cancers via H3K27me.^16^ EZH2 expression is elevated through the regulatory effects of various microRNAs and lncRNAs (long noncoding RNAs), thereby promoting the development of digestive cancers. For example, EZH2 is recruited by the lncRNA UCA1 and promotes direct interaction with the cyclin D1 promoter to activate the translation of cyclin D1, thereby fueling tumor growth in GC.^17^ Loss of miR-355 upregulates EZH2 expression by activating Sox4 in PC.^18^ In addition, loss of miR-26, miR-101, and miR-214 contributes to overexpression of EZH2, leading to the development of digestive cancers.^19–23^ Among the abovementioned microRNAs, the effect of miR-26 on EZH2 has been widely noted and has been identified in HCC, CRC, and GC.^19,20,22^ Another study identified that EZH2 can also bind to the lncRNA HOX-AS2 together with KDM1A to form a complex implicated in PC cell proliferation,^24^ and a similar function can also be performed by the lncRNA HOXA11-AS in GC.^25^ In addition to being modulated by microRNAs and lncRNAs, the expression of EZH2 is also promoted by cyclin-dependent kinase 4 (CDK4),^26^ Snail,^27^ Yin Yang-1 (YY-1),^28^ proviral integration site 1 (PIM-1) kinases,^29^ damage specific DNA-binding protein 2 (DDB2),^30^ nonacetylated homeobox B9 (HOXB9),^31^ and signal transducer and activator of transcription 3 (STAT3) signaling,^32^ thus contributing to the malignancy of digestive cancers. However, the activation of EZH2 may not be that straightforward; Battistelli et al. reported that the EMT-inducing transcription factor (EMT-TF) Snail recruits EZH2 to its target gene promoters in a HOTAIR-dependent manner,^27^ indicating that EZH2 activation is actually quite a complex process needing further exploration. Furthermore, the catalytic activity of EZH2 is not only determined by its own expression but also affected by its cofactors in PRC2. For example, activation of another core protein component of PRC2, embryonic ectoderm development (EED), promotes the catalytic function of EZH2, resulting in increased levels of H3K27me3 in PC cells.^33^ In addition, other studies have demonstrated that H3K27me3 levels in cancer cells are decreased concomitant with the reduction in the expression of suppressor of zeste 12 (SUZ12 protein homolog, a core component of PRC2), inhibiting the development of HCC and GC.^34,35^

KMT2A (also called mixed lineage leukemia 1, MLL1) is another histone methyltransferase attracting increasing attention in digestive cancers. KMT2A functions by forming the MWRAD complex with WD repeat domain 5 (WDR5), RBBP5, ASH2L, and DPY30,^36^ which possesses H3K4 methyltransferase activity and results in the installation of activating marks on chromatin at actively transcribed regions. Analysis of genomic and epigenomic features of HCC showed that p53 mutation led to independent transcriptional activation that could directly regulate a distinct set of chromatin regulators, including KMT2A.^37^ In addition, KMT2A expression is reduced by a decrease in the level of hPaf1/PD2 level, which decreases H3K4me2/3 and induces a corresponding decrease in the level of chromohelicase DNA-binding protein 1 (CHD1), an ATP-dependent chromatin remodeling enzyme that specifically binds to H3K4me2/3 marks.^38^ In addition to the influence of KMT2A targeting on MWRAD complex activity, the regulation of other components of the complex also affects its catalytic function.^39^ Studies have demonstrated that knockdown of WDR5 impacts H3K4 methylation in CRC cells and PC cells.^40,41^ Activation of WDR5 by the lncRNAs GCAWKR and GClnc1 facilitates H3K4me3 modification of the target genes protein tyrosine phosphatase type IVA (PTP4A1, member 1) and superoxide dismutase 2 (SOD2, mitochondrial) in GC.^42,43^ Furthermore, the lncRNA HOTTIP promotes HOXA9 expression by binding WDR5 to enhance Wnt/β-catenin pathway signaling, thus augmenting cancer stem cell (CSC) properties in human PC.^44^ Meanwhile, a study conducted by Mishra et al. has shown that KMT2A promotes leukemogenesis mainly through recruitment of MOF and forming H4K16ac (acetylation of lysine 16 of histone 4), not the intrinsic histone methyltransferase activity of KMT2A. However, the role of this mechanism in digestion cancers still needs further exploration.^45^

### KDMs

KDMs are a class of enzymes that play a role in the demethylation of histone lysines. Similar to KMTs, KDMs are diverse, but recent whole-genome sequencing and copy number analysis identified KDM6A (lysine demethylase 6A) as one of the most frequently altered histone methylation modifiers in digestive cancers.^13,46–49^ KDM6A, also called ubiquitously transcribed X (UTX chromosome tetratricopeptide repeat protein), is a Jumonji C (JmjC) domain-containing demethylase that targets H3K27 in complex proteins associated with Set1 (COMPASS), contributing to transcriptional activation.^50,51^ In solid pseudopapillary neoplasms (SPNs), which are borderline pancreatic tumors, recruitment of KDM6A to the enhancer of the SCL2A1 gene is inhibited due to deletion of BRCA1 associated protein-1 (BAP1),^52,53^ thus activating SCL2A1 transcription and driving the malignancy of SPNs. In addition to performing its histone methylation function alone, KDM6A can also interact with other histone modification enzymes. For example, KDM6A interacts with the histone acetyltransferase protein CBP and recruits it to the CDH1 (E-cadherin) promoter in HCT-116 CRC cells, resulting in increased H3K27ac, a transcription-activating histone mark.^54^ Similarly, during PC, KDM6A cooperates with the histone acetyltransferase p300 to regulate gene expression, while loss of KDM6A results in decreased H3K27ac levels in the promoter regions of tumor suppressor genes such as CDKN1A, LOXL1, SASH1, TXNIP, and IGFBP2.^55^ In addition, another study showed that loss of KDM6A rewires the enhancers and/or activated super-enhancers (SEs) of several tumor-related genes,^56^ which are formed by clusters of H3K27ac-marked enhancers.^57,58^

In addition to KDM6A, other KDMs are activated or inhibited under certain conditions, thus playing a corresponding role in digestive cancers. PHD finger protein 5A (PHF5A) K29 acetylation enhances KDM3A expression by stabilizing its mRNA. PHF5A acetylation increases the level of the H3K9me1/2 demethylase KDM3A by reducing its aberrant alternative splicing of its mRNA in CRC, thereby modulating stress responses and carcinogenesis in CRC.^59^ KDM3A can also be activated by HIF1α via co-activators that bind to hypoxia response elements (HREs) in the doublecortin calmodulin-like kinase 1 (DCLK1) gene under hypoxic conditions in PC;^60^ DCLK1 is a marker of CSCs, and this process is promoted by protein inhibitor of activated STAT protein 4 (PIAS4).^61^ RUNX family transcription factor 3 (RUNX3)-mediated upregulation of miR-29b increases its targeting of the KDM2A 3′-UTR and decreases KDM2A expression, which suppresses the proliferation and migration of GC cells.^62^ Furthermore, KDM4A is activated by loss of miR-137,^63^ and KDM4C is activated by CircZMYM2 via its sponging of miR-335-5p in PC.^64^ Both KDM4A and KDM4C target H3K9me2/3, H3K36me2/3, and H3K56me2/3. Sun et al. also found that the lncRNA HOXA11-AS can recruit KDM1A (an H3K4me1/2 and H3K9me1/2 demethylase) and functions as a scaffold for KDM1A, EZH2, and DNA-methyltransferase 1 (DNMT1) in GC, resulting in the progression of cancer development.^25^ Moreover, KDMs are closely related both to each other and to other histone modification enzymes. For example, Tzatsos et al. found that KDM2B contributes to overexpression of KDM5A and EZH2, while KDM2B interacts with KDM5A and MYC to activate metabolic and ribosomal gene expression and binds EZH2 to silence the expression of lineage specification genes.^65^

### PRMTs

Histone arginine methylation, which is catalyzed mainly by PRMTs, is also important in digestive cancers, but reports on histone arginine methylation are more scarce than those on histone lysine methylation to date. PRMTs can be divided into three categories according to their catalytic activity: type I (PRMT1, PRMT2, PRMT3, PRMT4, PRMT6, and PRMT8), type II (PRMT5 and PRMT9) and type III (PRMT7). All PRMTs use S-adenosylmethionine (SAM) as the methyl donor to catalyze three major forms of arginine methylation on histones: monomethylarginine (MMA), asymmetric dimethylarginine (aDMA), and symmetric dimethylarginine (sDMA).^66^

Multiple studies have demonstrated that several PRMTs, such as the histone H3 arginine 8 (H3R8) and H4R3 methyltransferase PRMT5,^67,68^ and histone H3 arginine 2 (H3R2) methyltransferase PRMT6,^69,70^ are highly expressed in digestive cancer cells. During the development of colon cancer, intestine-specific peroxisome proliferator-activated receptor alpha (PPARa) deficiency promotes PRMT6 expression via the RB1/E2F pathway and increases the enrichment of H3R2 asymmetric dimethylation (H3R2me2a) in the promoter of p27.^70^ Another study demonstrated that PRMT9 promotes HCC invasion and metastasis by activating PI3K/Akt/GSK‐3β/Snail signaling.^71^ However, the study did not explain whether this activation was caused by histone methylation of related genes, and this possibility needs further investigation. In addition, PRMTs not only play a role through histone arginine methylation but can also be associated with other histone methyltransferases. For example, recent studies have demonstrated that upregulated PRMT5 can epigenetically silence the expression of the E3 ubiquitin ligase F-box and WD repeat domain-containing 7 (FBW7) by increasing H4R3me2s and H3K9me3 and decreasing H3K9ac in PC,^72^ indicating that PRMT5 can recruit histone KMTs and histone deacetylases to promote tumor development. This process results in c-Myc stabilization,^72^ and fibroblast growth factor-binding protein 1 (FGFBP1) is a downstream target of the FBW7/c-Myc axis; thus, the FGFBP1-mediated FGF signaling pathway may be involved in the process described above.^73^ However, in addition to the histone methylation function of PRMTs, their non-histone methylation function has also received increasing attention. For instance, PRMT3-mediated methylation of glyceraldehyde-3-phosphate dehydrogenase (GAPDH) and PRMT4-mediated methylation of malate dehydrogenase 1 (MDH1) are involved in the metabolic reprogramming and proliferation of PC cells.^74,75^

## Histone methylation mediates the development of digestive cancers

### Carcinogenesis

Under stimulation by some external or internal factors, the regulation of histone methylation of cancer-related genes in normal digestive system cells may lead to abnormal cell growth and differentiation and thus to the occurrence of digestive cancer.

Hepatitis caused by viruses, alcohol, or fat intake is a major cause of HCC, and histone methylation plays an important role in its progression. Fan et al. demonstrated that in mice, KMT1B-mediated H3K9me3 contributes to nonalcoholic steatohepatitis, which accelerates hepatitis-induced hepatocarcinogenesis. In hepatocytes, KMT1B-mediated H3K9me3 represses Sirt1 transcription, while in macrophages, KMT1B-mediated repression of PPARγ transcription favors the proinflammatory M1 phenotype over the anti-inflammatory M2 phenotype, thereby elevating hepatic inflammation.^76^ Moreover, chromatin immunoprecipitation (ChIP) assays showed that during hepatitis B virus-induced hepatocarcinogenesis, HBV X protein (HBx) expression decreases the level of the silencing modification H3K27me3, while the level of the activating histone modification H3K4me1 is increased in the promoter of EpCAM, a host gene involved in HBV-mediated hepatocarcinogenesis.^34^ Similar conclusions were reached in a subsequent study conducted by Zhang et al.^77^

Regarding gastrointestinal carcinogenesis, researchers have found that KDM4B physically interacts with c-Jun on the promoters of IL-8, MMP1, and ITGAV via its demethylation activity, while infection with *Helicobacter pylori* significantly increases the occupancy of KDM4B and c-Jun, resulting in a significantly diminished H3K9me3 signal.^78^ Furthermore, another study identified three H3K27me modifier genes (EZH2, KDM6A, and KDM6B) that are individually associated with GC susceptibility via a synergistic triad interaction.^46^ During human colorectal carcinogenesis, mutations in Wnt/β-catenin signaling mediators may be one of the earliest events that initiates and drives tumor progression.^79^ Upregulation of disheveled segment polarity protein 22 (DVL), resulting from a significant decrease in H3K36me3 in the absence of KMT3A, leads to enhancement of Wnt/β-catenin signaling pathway activity and thereby drives colorectal carcinogenesis. Moreover, another study revealed the decrease in H3K27me3 and the increase in H3K4me3 in the WNT3 promoter region, indicating that histone methylation directly activates the Wnt/β-catenin signaling pathway to promote the initiation of CRC.^80^

In the pancreas, histone methylation plays a crucial role in the growth and differentiation of pancreatic cells even under physiological conditions. Studies have shown that KDM6A/B are crucial in endoderm differentiation in the pancreas.^81,82^ At the early stage of definitive endoderm differentiation, KDM6A/B promote mesoderm differentiation by demethylating H3K27me3, which upregulates WNT3 expression and activates the WNT signaling pathway. At the late stage, KDM6A/B demethylates H3K27me3 to activate the WNT antagonist DKK1 in order to suppress the WNT signaling pathway and promote endoderm differentiation over mesoderm differentiation.^82^ However, under some conditions, such as injury to the pancreas or chronic inflammation, loss of EZH2 results in impaired pancreatic regeneration and accelerates KrasG12D-driven neoplasia.^83^ Further studies showed that EZH2 is upregulated in injured pancreatic cells, while the presence of KRAS reverses the function of EZH2 during late-stage regeneration, thus sustaining the expression of nuclear factor of activated T cells 1 (NFATc1), a crucial driver of pancreatic carcinogenesis and progression, through a decrease in H3K27me3 and increase in H3K4me3.^84^ This event integrates signals from inflammatory networks to control cellular plasticity and counteracts TGFβ-mediated apoptosis induction.^85–87^

Acinar-to-ductal metaplasia (ADM), which is essential for PC carcinogenesis, is a reprogramming event in which acinar cells display marked cellular plasticity and transiently transform into progenitor-like cells exhibiting a ductal morphology in pancreatitis.^88,89^ This process, in conjunction with the expression of oncogenes such as K-ras, may trigger the development of PC.^90^ ChIP-seq data showed a significant gain of H3K27me3 and H2AK119ub (ubiquitination of lysine 119 of histone 2) at the regulatory acinar cell fate genes RBPJL, PTF1A, NR5A2, and BHLHA15 in pancreatic cells that had undergone ADM, and additional significant loss of H3K4me3 on these genes was found in PC cells.^91^ In addition, H3K4me3 on the Sox9 promoter increases as a result of NFATc1 activation during the ADM process, resulting in elevated expression of Sox9, a downstream effector of oncogenic Kras, and accelerates the formation of premalignant lesions in the pancreas.^92,93^

### Proliferation

Uncontrolled proliferation is an important characteristic of tumor cells. Under appropriate conditions, cancer cells can multiply indefinitely and become immortalized.^94^ Histone methylation plays a key role in the uncontrolled proliferation of digestive cancer cells. For example, repressive H3K27me3 marks installed by EZH2 are enriched in the Kruppel-like factor 2 (KLF2) promoter, leading to a growth advantage in the tumor cell population in GC and CRC,^95,96^ via the HIF-1α/Notch-1 signaling pathway and the Hedgehog pathway.^97,98^ In addition, the proliferation of CRC cells is significantly accelerated by the EZH2-mediated H3K27me3 modification of the dual specificity phosphatase 5 (DUSP5) gene,^99^ a negative regulator of the mitogen-activated protein kinase (MAPK)-signaling pathway.^100^

### Cell cycle dysregulation

Abnormalities in cell cycle network are closely related to the proliferation of tumor cells. During the development of digestive cancers, histone methylation can regulate cell cycle by regulating cyclins, CDKs cyclin-dependent kinase inhibitors (CKIs), thus causing cell cycle dysregulation and uncontrolled cell proliferation. Previous experiments with human pancreatic cell lines showed that KMT2D silencing leads to a decrease in the number and proportion of cells in G0/G1, accompanied by a global reduction in H3K4me1/2/3,^101^ suggesting that histone methylation is indeed involved in cell cycle regulation. Further studies have focused mainly on the regulation of CKIs.

The genes encoding P15 and P21, two commonly mentioned CKIs, exhibit increased levels of H3K27me3 and H3K9me3 and decreased levels of H3K4me2/3 in digestive cancers, including GC,^102–104^ CRC,^105,106^ HCC,^107,108^ and PC.^109^ This process, triggered by upstream lncRNAs such as BLACAT1, SNHG17 and CASC15, can inhibit the expression of P15 and P21 and lead to G0/G1 checkpoint deficiency.^110–112^ In addition, Zhang et al. found that EZH2-mediated H3K27me3 downregulates the expression of the p57 gene in addition to p15 gene, contributing to the regulation of the GC cell cycle and GC cell proliferation.^113^ In addition, TWIST recruits EZH2 to induce H3K27me3, which represses the transcription of the p16 gene by binding to the E-box in its promoter in PC cells under hypoxic conditions,^114^ and the same modification can be initiated by the lncRNA PVT1 in GC.^115^ P27kip1 is similarly inhibited by EZH2-mediated H3K27me3; the only difference is that EZH2 binds and modifies exon 1 rather than the promoter region.^116^ Another study suggested that EZH2 induced by KRAS mutation installs H3K27me3 in the promoter of miR-34a, thus epigenetically inactivating miR-34a expression to suppress the effects of p53 transactivation.^117^

Similarly, histone methylation also regulates cyclin and CDK in digestive cancer cells. According to recent studies, Jumonji domain-containing protein Jumonji domain-containing protein 6 (6JMJD6), a histone arginine demethylase, activates CDK4 expression by decreasing the H4R3me2s level in the CDK4 promoter in HCC.^118^ In addition, the transcription of CDK2 is promoted by KMT3E-mediated increases in H3K4me3 enrichment at the corresponding promoter sites.^119^ Regarding cyclins, the level of cyclin D1 is significantly increased due to decreases in repressive H3K9me2 marks and increases in activating H3K4me3 marks installed by KDMA/B and KMT2A, respectively.^120^ In addition, upregulation of Forkhead box transcription factor M1 (FOXM1) by KMT4-induced H3K79me2 in PC significantly increases the expression of its classical target genes cyclin A2 and cyclin B1 through the Wnt5a signaling pathway.^121^

### Immune escape

In addition to the uncontrolled proliferation caused by cell cycle dysregulation, abnormal negative regulatory mechanisms of cell proliferation, especially immune escape, can also cause abnormal proliferation during the development of digestive cancers. For example, researchers have demonstrated that during PC, H3K4me3 in the BCL2L1, CFLAR, and MCL-1 gene promoters upregulates the expression levels of the anti-apoptotic proteins Bcl-x, FLIP, and Mcl-1 and that H3K9me3 in the BAK1, BAX, and BCL2L11 gene promoters downregulates the expression levels of the pro-apoptotic proteins Bak, Bax, and Bim.^122^ All six of these apoptosis-regulating genes are involved in PC growth and progression.^123–126^ In addition, a genome-wide ChIP-sequencing analysis identified that H3K9me3 is enriched in the FAS promoter in metastatic human colon carcinoma cells; this enrichment inhibits the expression of Fas and results in resistance to Fas-mediated apoptosis.^127^ Furthermore, EZH2-mediated H3K27me3 by the lncRNA HOTAIR in the death receptor 5 (DR5) gene promoter results in resistance to TNF-related apoptosis-inducing ligand (TRAIL)-induced apoptosis;^128^ the same modification is found in the proapoptotic genes growth differentiation factor 15 (GDF15) and KLF2.^129,130^

In addition to apoptosis resistance, the production of the Th1-type chemokines CXC chemokine ligand 9 (CXCL9) and CXCL10, mediators of effector T cell trafficking, is suppressed by H3K27me3 in their gene promoters in colon cancer.^131^ However, the expression of CXC chemokine receptor 4 (CXCR4) is promoted by EZH2-mediated loss of miR-622,^132^ and CXCR4 can facilitate the evasion of immune surveillance by binding to CXCL12.^133^ Another experiment conducted by Bugide et al. showed that overexpressed EZH2 associates directly with the promoters of natural killer (NK) cell ligands, such as ULBP1 and MICA in HCC cells and then catalyzes the installation of repressive H3K27me3 marks on these promoters.^134^ In addition, Zhou et al. found that in PC, upregulation of FOXM1 by KMT4-induced H3K79me2 significantly attenuates antitumor responses, including bone marrow-derived dendritic cell (BMDC) maturation, cytokine secretion, and T cell activation, through the Wnt5a signaling pathway.^121^ Moreover, in PC cells, KMT2A overexpression increases the H3K4me3 level in the CD274 promoter region and upregulates the expression of PD-L1,^135^ a T cell inhibitory receptor ligand that results in potent immunosuppression.^136^

### Metabolic reprogramming

To support the ability for uncontrolled proliferation, tumor cells have adapted many aspects of their metabolic patterns. One of the most well-known examples is the Warburg effect, through which the rate of aerobic glycolysis is increased in cancer cells, thus leading to decreased glucose oxidation and enhanced flux through the anabolic side branches of glycolysis.^137^ Similarly, the Warburg effect can be induced in digestive cancer cells through histone methylation or demethylation of related genes. Glucose transporter 1 (GLUT1), one of the major components regulating glucose homeostasis,^138^ can be activated by p-ERK/KDM4B-mediated removal of the repressive H3K9me3 mark, thus contributing to glucose uptake in colon cancer cells under glucose deprivation conditions.^139^ KMT2D inhibition mediates the loss of H3K4me3 in metabolic pathway genes, which results in alterations in aerobic glycolysis and lipid levels via GLUT3-mediated processes.^140^ As GLUT3 exhibits a high affinity for glucose, thus ensuring efficient glucose uptake into cancer cells,^141^ this process induces aerobic glycolysis and increases the levels of lipids, such as docosadienoic acid, docosatrienoic acid, and docosatetraenoic acid, which harbors oncogenic properties in PC cells.^140^ In addition, Sakamoto et al. demonstrated that H3K4me2 demethylation mediated by KDM1A overexpression suppresses the expression of two metabolism-related genes, PGC-1α and LCAD, which are involved in the transcriptional control of genes related to mitochondrial oxidative metabolism and fatty acid oxidation, respectively.^142^ Similar modifications can occur on the glyceronephosphate O-acyltransferase (GNPAT) gene to activate its transcription; the only difference is that this process is triggered by c-Myc. GNPAT recruits the enzyme USP30, which deubiquitylates and stabilizes dynamin-related protein 1 (DRP1), thus promoting dysregulation of mitochondrial morphology and lipid metabolism in HCC.^143^ Moreover, KDM5A-mediated H3K4me3 demethylation of the mitochondrial pyruvate carrier 1 (MPC-1) gene transcriptionally inhibits its expression, leading to elevated mitochondrial pyruvate metabolism and inhibition of glycolysis. As a result, a series of metabolic disorders occur, such as hyperpyruvatemia and lactic acidosis.^144^ In addition, another study confirmed that cooperative binding of KDM2B, KDM5A, and/or MYC activates the expression of genes involved in metabolic homeostasis and protein synthesis, such as ATP5G2 and RPL3/7/24/30, via enrichment of H3K4me3 on these genes.^65^

Regulation of histone methylation can affect metabolism in digestive cancer cells, and the metabolites in digestive cancer cells can reciprocally affect histone methylation. For example, FH (fumarase), which catalyzes the reversible hydration and dehydration of fumarate, can bind to the c-Jun gene promoter, inhibiting KDM2A activity through local fumarate production and promoting H3K36me2.^145^ In addition, Oliver et al. demonstrated that 6-aminonicotinamide (6AN), a 6-phosphogluconate dehydrogenase (PGD) inhibitor, induces broad enrichment of H3K9me2 across the CDH2 (N-cadherin) and topoisomerase 2β (TOP2B) genes, causing distant metastasis of PC.^146^

Interestingly, collectively, these studies suggest that histone methylation and metabolic reprogramming may form a positive feedback loop to promote digestive cancer progression. For instance, Yi et al. reported that HIF1a stabilization under hypoxic conditions leads to KDM1A upregulation at the mRNA and protein levels in PC,^147^ while progression of PC induces a hypoxic microenvironment through the Warburg effect and results in the production of a large amount of HIF1.^148,149^ A recent study conducted by Jiang et al. in CRC also supported this conclusion. O-linked N-acetylglucosamine transferase (OGT), which is negatively regulated by miR-101, enhances EZH2 protein stability and function and promotes the enrichment of repressive H3K27me3 marks on the miR-101 promoter region, resulting in the upregulation of OGT.^150^ This process creates a vicious cycle and further exacerbates tumor progression.

### EMT

EMT is an important pathophysiological event that results in the loss of cell–cell adhesion, abnormal apical–basal polarity, and cytoskeletal reorganization, thereby enabling polarized, immotile epithelial cells to acquire mesenchymal abilities such as invasiveness and motility.^151^ Accumulating data have indicated that EMT leads to enhanced cell invasion and migration in digestive cancers.^152,153^ Moreover, the results of a tag and track experiment in pancreatic epithelial cells conducted by Rhim et al. indicated that EMT can occur even before the formation of cancer, showing that pancreatic epithelial cells can invade and enter the bloodstream to become circulating epithelial cells (CECs), maintaining the mesenchymal phenotype in pancreatic intraepithelial neoplasia (PanIN), a common precancerous lesion of PC.^154^ Several histone methylation modifications are involved in EMT in digestive cancers, especially EZH2-mediated H3K27me3 on the E-cadherin gene promoter. For example, Zhou et al. found that loss of ten-eleven translocation 1 (TET1) activates EZH2, which represses E-cadherin gene transcription by catalyzing H3K27me3 modification in the E-cadherin promoter in CRC cells.^155^ This modification leads to a decline in the expression of E-cadherin, a well-known hallmark of EMT.^156^ The same CDH1 gene promoter modification in CRC was also found in two other studies; one study showed that this modification was triggered by the lncRNA SNHG6^22^ and the other showed that it was induced by the EMT-TF Snail2.^157^ During PC, EZH2 can also be recruited by TWIST and loss of microRNA-101, which binds to one or more E-boxes in the E-cadherin gene promoter and increases repressive H3K27me3 marks.^114,158^ In addition, EZH2-mediated H3K27me3 can regulate EMT indirectly through microRNAs and lncRNAs. EZH2 epigenetically silences miR-34a through H3K27me3 enrichment in the miR-34a promoter region. Downregulation of miR-34a in turn activates c-Met and thus results in transcriptional activation of Snail, thus contributing to the EMT process in GC cells and accelerating tumor metastasis.^159^ Another study demonstrated that miR-34a, along with miR-203, is repressed by EZH2-mediated H3K27me3, thereby activating the Snail1 and Snail2 EMT-TFs to downregulate E-cadherin.^160^ MiR-139-5p transcription is inhibited by EZH2 through an increase in H3K27me3; therefore, the levels of the EMT-TFs ZEB1 and ZEB2 increase, while the E-cadherin level decreases.^161^

In addition to EZH2-mediated H3K27me3, other histone methylation modifications are involved in regulating EMT in digestive cancers. Loss of KDM6A negatively regulates E-cadherin expression by coordinating the regulation of H3K27 methylation in the E-cadherin promoter in HCT-116 colon cancer cells, switching the transcriptionally active state to a transcriptionally repressive state.^54^ In addition, G9a-mediated upregulation of H3K9me2, a heterochromatic histone methylation marker, has been observed at the same site in HCC cells and results in further inhibition of E-cadherin expression.^162,163^ Moreover, recent studies showed that ChIP assays can detect decreases in H3K9me3 and increases in H3K4me3, which are catalyzed by KDM4B and KMT7, respectively, in the ZEB1 promoter region.^164,165^ Meanwhile, KMT5C silences expression of the mesenchymal-to-epithelial transition (MET)-promoting transcription factors FOXA1, OVOL1, and OVOL2 via its repressive mark H4K20me3 in PC.^166^ Furthermore, PRMT1 can bind to the promoter region of CTNNB1 and increase the β-catenin protein level in PC cells,^167^ but whether this effect is related to histone methylation needs further exploration.

### Invasion and migration

Invasion and migration of cancer cells into the surrounding tissue and vasculature is an essential initial step in cancer metastasis, which contributes to the terminal stages of cancer. During this process, digestive cancer cells invade deep tissue and enter lymphatic and blood vessels for dissemination into the circulation via regulation of histone methylation. This dissemination enables the cancer cells to colonize distant organs.

For example, decreases in H3K27me3 in the MMP7 and HMGA2 promoter regions promote the progression of PC in Brg1-depleted PC cells,^168^ as MMP7 facilitates tumor cell invasion and HMGA2 promotes invasion and migration through EMT.^169,170^ Moreover, two other MMP family proteins, MMP9 and MMP14, are also activated during the development of digestive cancers. The level of H3K9me3 in the MMP9 promoter is markedly decreased,^171^ while MMP14 exhibits robust accumulation of H3K4me3 and loss of H3K27me3.^91^

In addition to MMP family proteins, researchers have also found that ectopic expression of a cluster of genes that participate in tumor invasion and metastasis in the digestive system is associated with abnormal regulation of histone methylation. For instance, the invasion and migration abilities of cancer cells are promoted by H3K9me3-mediated epigenetic silencing of numerous related genes, such as IGFBP3, CXCL3, NOS3, and SLIT1.^172,173^ The opposite modification, demethylation of H3K9me3, occurs in the promoter of MALAT1, thereby upregulating the expression of MALAT1 and enhancing the activity of the β-catenin signaling pathway.^174^ In addition to H3K9 modification, decreases in H3K4me3 in BMP7, WIF1, and TIMP2/3 also significantly enhance the invasion and migration abilities of cancer cells in HCC and CRC,^175,176^ thus accelerating tumor progression. H3K27me3 also participates in this process. Tang et al. proved that aberrant KDM6B expression decreases H3K27me3 in the SLUG gene promoter and activates the transcription of SLUG, which promotes migration, invasion, and stem cell-like behaviors in HCC.^177^ Similar modifications can be detected in the Wnt10b promoter region in HCC; these modifications activate the Wnt/β-catenin signaling pathway and reinforce cell proliferation, migration, and invasion.^178^ Moreover, PRMT6-overexpressing GC cells also acquire invasiveness through direct transcriptional inhibition of PCDH7 by increasing H3R2me2as level.^179^ Another study conducted by Li et al. demonstrated that H3K27me3 and H3K9me2, mediated directly and indirectly by EZH2, are enriched in the miR-218-2 promoter, thus leading to downregulation of gene expression in PC. Loss of miR-218 increases invasion and migration through the effect of the UDP-glycosyltransferase 8 (UGT8) protein.^180^ In addition to EZH2, KDM6B is also involved in the processes of invasion and migration. Loss of this histone demethylase enhances the invasion and migration of PC cells through downregulation of CCAAT-enhancer-binding protein alpha (CEBPA); mechanistically, loss of KDM6B induces H3K27me3 in the region upstream of the CEBPA transcription start site.^181^

However, conflicting results have been obtained. For example, inconsistent with the above results, GDF15, a migration-promoting factor,^182^ was found to be downregulated in PC cells via a lncRNA HOTAIR-induced increase in the H3K27me3 level in its promoter.^129^ Moreover, researchers have proven that EZH2-mediated H3K27me3 suppresses RUNX3 gene expression in PC and GC.^183,184^ Notably, RUNX3 has traditionally been considered a tumor suppressor, but an article published in *Cell* in 2015 reported that it had a reinforcing effect on metastatic seeding and colonization via extracellular matrix (ECM) remodeling in PC.^185^ Therefore, in this case, inhibition of this suppressor reduces the malignant behavior of digestive cancer cells rather than promoting tumor development, indicating that EZH2 can function as both an oncogene and a tumor suppressor.

## Clinical application of histone methylation in digestive cancers

As mentioned above, the regulation of histone methylation has substantial clinical potential, especially in treatment strategies for digestive cancers. Currently, radical surgical resection is the most effective treatment. However, the prognosis of digestive cancers is still not very optimistic. In PC, for example, fewer than 20% of patients have the opportunity for surgery, and of those who undergo resection followed by adjuvant therapies, more than 80% relapse and ultimately die of their disease.^186^ Moreover, the lack of effective drugs for chemotherapy and targeted therapy and resistance to existing drugs are other important reasons for the dismal prognosis. For these reasons, discovering effective drug therapies for affected patients is of paramount importance. As discussed above, regulation of histone methylation plays a pivotal role in digestive cancer development. Thus, strategies that regulate histone methylation modifiers and thus control histone methylation may constitute potential treatments for digestive cancers.

### Histone methylation as an adjuvant to reverse drug resistance

Chemotherapy and targeted therapy are still the first-line treatment modalities for digestive cancers, including HCC,^187^ GC,^188^ CRC, and PC,^189–191^ but their therapeutic effect differs widely across patients. Studies have revealed that regulation of histone methylation plays an important role in determining the efficacy of and resistance to chemotherapy and targeted therapy;^192,193^ EZH2-mediated H3K27 methylation and KDM1A-mediated H3K4/9 demethylation^16,194^ are especially important. Lima-Fernandes et al. demonstrated that inhibition of the H3K27 methyltransferase EZH2 by UNC1999 resulted in increased sensitivity to 5-fluorouracil in CRC cells, accompanied by downregulation of H3K27me3 in the promoter of Indian Hedgehog and decreased self-renewal of CRC-initiating cells.^195^ In addition, metformin decreases the gene expression of EZH2 in pancreatospheres derived from gemcitabine-resistant PC cells, thus increasing the sensitivity of PC cells to gemcitabine via targeted killing of CSCs.^196^ Two other types of agents, lactate dehydrogenase A (LDHA) inhibitors and curcumin, have also recently been shown to display synergistic cytotoxic activity with gemcitabine in PC; the underlying mechanism is at least partially mediated by inhibition of EZH2.^197,198^

Suppression of KDM1A might be an attractive target for regorafenib sensitization and clinical HCC therapy, but an in-depth mechanistic investigation is lacking.^199^ Further research proved that KDM1A inhibitors, such as pargyline and GSK2879552, dramatically suppress the stem-like properties of sorafenib-resistant HCC cells, including resensitization to sorafenib. Mechanistically, these KDM1A inhibitors negatively regulate the Wnt/β-catenin pathway through enrichment of activating H3K4me1/2 marks in the promoter of the Wnt antagonists Prickle1, APC and Sfrp5.^200^ This finding is consistent with the results reported by Lei et al., which indicated that knockdown of KDM1A sensitizes HCC cancer cells to cisplatin and sorafenib by increasing the expression of several suppressors of β-catenin signaling, especially Prickle1 and APC.^201^

In addition to studies on targeting EZH2 and KDM1A, recent studies have demonstrated that small molecule inhibitors of KMT-4 (EPZ5676) combined with 5-fluorouracil or PARP inhibitors, which are used to treat CRCs, show additive effects accompanied by decreased H3K79me3 levels.^202^ Moreover, the G9a inhibitor UNC0638 notably enhances the cytotoxicity of topoisomerase-based treatment in CRC; mechanistically, H3K9me2 in the PP2A promoter is decreased, thus activating the PP2A–RPA axis.^203^ Verticillin A is a selective HMTase inhibitor that inhibits SUV39H1, SUV39H2, G9a, GLP, NSD2, and KMT2A, which decreases the H3K9me3 level in the FAS promoter and restores Fas expression, thus alleviating 5-fluorouracil resistance in CRC. Verticillin A is less toxic but exhibits greater efficacy than decitabine and vorinostat.^127^ Lu et al. proved that a sublethal dose of verticillin A effectively overcomes the resistance of human PC cells to gemcitabine and suppresses tumor growth.^122^ Furthermore, verticillin A was recently found to have a synergistic effect with anti-PD-L1 therapy in PC.^135^

### Histone methylation as a therapeutic target

In addition to reversing the effects of chemotherapeutic drug resistance, histone methylation can also constitute a direct therapeutic target in digestive cancers. In this regard, inhibition of EZH2-mediated H3K27me and G9a-mediated H3K9me/H3K56me are the most widely reported strategies. In fact, EZH2 inhibitors have even been identified as cancer prevention drugs. A recent study showed that suppressing EZH2 activity with GSK343 ameliorated experimental intestinal inflammation and delayed the onset of colitis-associated cancer accompanied by a gradual loss of H3K27me3 expression.^204^ DZNep (3-deazaneplanocin A), a potent chemical inhibitor of S-adenosylhomocysteine hydrolase that modulates chromatin accessibility through inhibition of histone methyltransferases, including EZH2,^205^ can lead to a significant reduction in H3K27me3 with a marked reduction in cell proliferation and migration in CRC. Similar effects can also occur in PC, decreasing the global H3K27me3 level and subsequently causing reexpression of miR-218, thus inhibiting cell proliferation, promoting apoptosis, and inducing cell cycle arrest in PC cells.^180^ A later study found that DZNep significantly alters miR-663a and miR-4787-5p expression and suppresses TGFb1-induced EMT signaling in PC.^206^ GSK126 is another EZH2 inhibitor that leads to epigenetic reprogramming in cancer cells, thus successfully promoting the infiltration of functional CD8þ T cells and substantially suppressing HCC growth.^207^ In addition, Huang et al. reported that suppressing EZH2-mediated H3K27me3 with GSK126 results in increased numbers of myeloid-derived suppressor cells (MDSCs) and decreased numbers of CD4+ and IFN-γ+ CD8+ T cells, which are closely related to antitumor immunity in CRC.^208^ UNC1999, an EZH2-specific inhibitor, not only reduced the aberrant H3K27 methylation that characterizes PC cells but also slowed the proliferation of cancer cells in three model systems.^209^ In addition, chaetospirolactone has been demonstrated to suppress the activity of the epigenetic regulator EZH2 and consistently decrease H3K27me3 to allow the transcription of DR4,^210^ which binds to TRAIL and leads to activation of the initiator caspase-8 and assembly of the death-inducing signaling complex (DISC).^211^ Subsequently, diosgenin, garcinol, FBW7, and the curcumin analog CDF were also identified as potential agents targeting EZH2 and thus hindering the development of PC.^212–215^

Inhibition of not only EZH2 but also G9a counteracts the development of digestive cancers. For example, pharmacological knockdown of G9a activity triggers an increase in autophagy by decreasing histone H3K9me2 and increasing histone H3K9ac, which leads to elevated expression of autophagy genes, such as LC3B, WIPI1, DOR, and p62, in PC cells, ultimately decreasing cell viability.^7^ Another study conducted by Kim et al. showed similar results in GC cells, while G9a inhibition in GC was mediated by kaempferol.^216^ A novel G9a inhibitor, BRD4770, was discovered to reduce cellular levels of H3K9me2 and H3K9me3 without inducing apoptosis, to induce senescence through ATM pathway activation, and to inhibit proliferation in the PC cell line PANC-1.^217,218^ Moreover, recent research has led to innovations in drug administration. Nanodiamond-mediated delivery of the G9a inhibitor UNC0646 maintained the biological functionality of UNC0646, with improved efficacy in reducing H3K9 methylation as well as enhancing the suppression of invasion in HCC cells,^219^ laying the foundation for future administration of small molecule histone methylation-regulating drugs. Similarly, dual targeting of G9a and DNMT1 by compounds such as CM-272 has also been proposed, thus providing a new perspective on HCC treatment.^220^

In addition to EZH2 and G9a, the novel curcumin analog L48H37 downregulates the histone methyltransferase KMT2D, as shown in a recent study, and KMT2D deficiency augments L48H37-induced apoptosis and blocks migration,^221^ thus forming a positive feedback loop. Moreover, combination treatment with the pan-H3K9me HMT inhibitor chaetocin and an aurora kinase A (AURKA) inhibitor diminishes H3K9 methylation at centromeres, induces mitotic aberrations, triggers an abnormal mitotic checkpoint response, and ultimately leads to mitotic catastrophe in PC.^222^ At the same time, the prospect of arginine methylation as a therapeutic target has been revealed gradually. For example, a recent study found that targeting PRMT5 activity by DW47800 inhibits the malignancy of HCC by downregulating the binding of H4R3me2s to the HNF4α promoter.^223^ Meanwhile, AMI-1, a small molecule inhibitor of PRMTs, was demonstrated to strongly inhibit proliferation and migratory activity in HCC and GC, along with a decreased expression levels of H4R3me2s and H3R8me2s.^224,225^

## Conclusion

As stated above, increasing attention has been focused on the role of histone methylation in the development of digestive cancers and its potential in clinical application. However, current studies still have limitations. For example, EZH2 has been found to act as both an oncogene and a tumor suppressor, since it maintains, rather than specifies, the transcriptional repression state of thousands of target genes. EZH2 acts most often as a mediator in tumor development; thus, it may play different roles under different conditions.^226^ Furthermore, the mechanism by which histone methylation regulates gene expression is still controversial. In contrast to the traditional understanding, some researchers believe that H3K4me3 may be not the “cause” but rather the “effect” of upregulated gene expression. Demethylation of CpG islands may shape the distribution of H3K4me3, and, in turn, H3K4me3 may influence the chromatin landscape at CpG islands.^227,228^ Therefore, the detailed mechanism linking H3K4me3 to upregulation of gene expression remains to be explored.

In addition, most current studies have focused on histone lysine methylation, and the role of histone arginine methylation has been relatively neglected. The crosstalk between histone arginine methylation and lysine methylation is also important, and may play important roles in the development of digestive cancers. Since histone methylation can regulate gene expression, histone lysine methylation may occur on histone arginine methyltransferase genes to regulate histone arginine methylation, and vice versa. Demetriadou et al. reported that activating H3K4me3 marks are reduced but repressive H3K27me3 marks are significantly enriched in the PRMT5 gene promoter in HCT116 cells with depletion of N-alpha-acetyltransferase 40 (NAA40).^229^ Another study proved upregulation of PRMT5 induces increasing H4R3me2s and H3K9me3 in FBW7 gene in PC, indicating that PRMT5 can recruit histone KMTs and histone deacetylases to promote tumor development. These patterns suggest that interaction indeed occurs between histone arginine methylation and lysine methylation. Such interaction and internal mechanisms should be further investigated, such as the event upstream of this action and explanation of how histone modifiers are recruited. To broaden the research scope, the interactions among histone methylation, histone acetylation, DNA methylation, and other epigenetic modifications should also be given full attention.

Moreover, although new therapeutic targets and molecular mechanisms are being revealed, only a modest number of clinical trials have been conducted (Table 1). Furthermore, to date, clinical trials have focused primarily on the effects of histone methylation modifier inhibitors on hematologic malignancies, such as B-cell lymphoma and non-Hodgkin lymphoma. Most of the relevant clinical trials on digestive cancers are in the initial stages, most commonly phase 1 and phase 2. Moreover, clinical trials of EZH2 antagonists are far more numerous than those of other histone modification modifier inhibitors, such as tazemetostat and CPI-1205. Future studies should devote more attention to these aspects to identify novel treatment options for digestive cancers.

**Table 1:** Clinical trials targeting histone methylation modifiers in digestive cancers

Compound	Epigenetic targets	NCT number	Phase	Enrolled tumor entities	Status
Tazemetostat (EPZ-6438)	EZH2	NCT03028103	1	B-cell lymphoma or advanced solid tumor	Active, not recruiting
Tazemetostat (EPZ-6438)	EZH2	NCT01897571	1/2	Advanced solid tumors, B-cell lymphomas or follicular lymphoma	Active, not recruiting
Tazemetostat (EPZ-6438)	EZH2	NCT03213665	2	Relapsed or refractory advanced solid tumors, non-Hodgkin lymphoma, or histiocytic disorders	Recruiting
Tazemetostat (EPZ-6438)	EZH2	NCT03155620	2	Relapsed or refractory advanced solid tumors, non-Hodgkin lymphomas, or histiocytic disorders	Recruiting
Tazemetostat (EPZ-6438)	EZH2	NCT02875548	2	Diffuse large B-cell lymphoma, follicular lymphoma, rhabdoid tumors, synovial/epitheliod sarcoma, mesothelioma, advanced solid tumors	Recruiting
Tazemetostat (EPZ-6438)	EZH2	NCT04241835	1	Advanced malignant solid tumor	Recruiting
Tazemetostat (EPZ-6438)	EZH2	NCT02601950	2	INI1-negative tumors or relapsed/refractory synovial sarcoma	Recruiting
Tazemetostat (EPZ 6438)	EZH2	NCT03010982	1	B-cell lymphomas or advanced solid tumors	Completed
Tazemetostat (EPZ-6438)	EZH2	NCT03217253	1	Metastatic or unresectable solid tumors or B-cell lymphomas	Withdrawn
GSK2816126	EZH2	NCT02082977	1	Relapsed/refractory diffuse large B cell lymphoma, transformed follicular lymphoma, other non-Hodgkin’s lymphomas, solid tumors and multiple myeloma	Terminated
CPI-1205	EZH2	NCT03525795	1/2	Advanced solid tumors	Active, not recruiting

## Supplementary Information

Supplementary Information
